# Electrospun Composite Nanofiltration Membranes for Arsenic Removal

**DOI:** 10.3390/polym14101980

**Published:** 2022-05-12

**Authors:** Tawsif Siddique, Rajkamal Balu, Jitendra Mata, Naba K. Dutta, Namita Roy Choudhury

**Affiliations:** 1 Chemical and Environmental Engineering, School of Engineering, RMIT University, Melbourne, VIC 3000, Australia; s3642366@student.rmit.edu.au (T.S.); rajkamal.balu@rmit.edu.au (R.B.); 2 Australian Centre for Neutron Scattering (ACNS), Australian Nuclear Science and Technology Organisation (ANSTO), Lucas Heights, NSW 2232, Australia; jitendra.mata@ansto.gov.au

**Keywords:** polysulfone, graphene oxide, zinc oxide, electrospinning, mixed matrix membrane, nanofiltration, arsenic, SANS

## Abstract

In recent years, significant attention has been paid towards the study and application of mixed matrix nanofibrous membranes for water treatment. The focus of this study is to develop and characterize functional polysulfone (PSf)-based composite nanofiltration (NF) membranes comprising two different oxides, such as graphene oxide (GO) and zinc oxide (ZnO) for arsenic removal from water. PSf/GO- and PSf/ZnO-mixed matrix NF membranes were fabricated using the electrospinning technique, and subsequently examined for their physicochemical properties and evaluated for their performance for arsenite–As(III) and arsenate–As(V) rejection. The effect of GO and ZnO on the morphology, hierarchical structure, and hydrophilicity of fabricated membranes was studied using a scanning electron microscope (SEM), small and ultra-small angle neutron scattering (USANS and SANS), contact angle, zeta potential, and BET (Brunauer, Emmett and Teller) surface area analysis. Fourier transform infrared spectroscopy (FTIR), X-ray diffraction (XRD), and X-ray photoelectron spectroscopy (XPS) were used to study the elemental compositions and polymer-oxide interaction in the membranes. The incorporation of GO and ZnO in PSf matrix reduced the fiber diameter but increased the porosity, hydrophilicity, and surface negative charge of the membranes. Among five membrane systems, PSf with 1% ZnO has the highest water permeability of 13, 13 and 11 L h^−1^ m^−2^ bar^−1^ for pure water, As(III), and As(V)-contaminated water, respectively. The composite NF membranes of PSf and ZnO exhibited enhanced (more than twice) arsenite removal (at 5 bar pressure) of 71% as compared to pristine PSf membranes, at 43%, whereas both membranes showed only a 27% removal for arsenate.

## 1. Introduction

Arsenic (As) is a natural element present in the earth crust and is listed as a major public health concern by the World Health Organization (WHO) [1]. Several unavoidable natural phenomena such as geological leaching and anthropogenic activities influence the level of As contamination in groundwater [2,3]. Long-time exposure to As can cause several health issues such as cardiovascular disorders, dermatological diseases, neurological disorders, and even cancer [4]. The As concentration in ground water is in the range of 1–100,000 μg/L. Around 150 million people in different parts of the world such as Bangladesh, China, India, and most South American countries are exposed to As concentration above the drinking water guidelines (50 μg/L) [5].

In recent years, different As treatment technologies such as adsorption, precipitation, coagulation ion-exchange, and membrane filtration have been studied [6,7,8]. Currently, membrane filtration is overpowering other methods because of low chemical consumption, zero by-products, easy scale-up, design simplicity, and easy operation [8,9,10]. Pressure-driven nanofiltration (NF) can be very efficient for As removal [11]. NF membranes are being used for different separation purposes because of their several advantageous characteristics, such as low energy consumption, high permeance, and molecular scale cut off [12]. Most used NF membranes are polyamide thin film composite membranes on microporous support. However, the polyamide NF membranes are prone to fouling and exhibit less stability towards chlorine [13,14,15].

To date, several polymers such as polysulfone (PSf), polyvinylidene fluoride (PVDF), polyethersulfone (PES), and bromomethylated poly(phenylene oxide) (BPPO) have demonstrated impressive performance as membrane materials for the ultrafiltration membrane [16,17,18]. However, these kinds of polymers are prone to fouling because of hydrophobicity, which leads to decreasing lifespan of the membrane [19,20,21]. Therefore, there is a drive to improve the hydrophilicity of these polymer membranes by a different approach such as incorporating nanoparticles, blending and/or grafting with hydrophilic polymer [22,23,24], and surface coating [25,26]. Among them, the incorporation of nanoparticles approach is widely practiced for improving hydrophilicity because of the simplicity and reproducibility of the process. 

PSf is a favorable polymer for making nanofiltration membranes because of its robustness, chemical and thermal stability, durability, and excellent pH tolerance [27]. However, it also comes with an undesirable fouling tendency because of its hydrophobicity. Therefore, membrane modification is taken into consideration for fouling control. Numerous nanoparticles have been used to prepare the mixed matrix membranes, which help to decrease the fouling and eventually increase the lifetime of the membrane [28]. The most popular filler material for modification of polymer membrane is carbon nanotube (CNT) [29], zirconium oxide (ZrO) [30], titanium oxide (TiO_2_) [24], silicon oxide (SiO_2_) [31], zinc oxide (ZnO) [32,33], zeolite [34], metal–organic frameworks (MOFs) [35], graphene oxide (GO) [36] for improving the surface hydrophilicity, and pore structure and distribution. 

ZnO is cheap and has demonstrated significant antibacterial and antifungal activity. In the last decade, several studies have reported the improvement of the hydrophilicity and anti-fouling activity of polymeric membranes by blending with ZnO [37,38]. ZnO nanoparticle is a zero-dimensional (0D) material, and a good inorganic filler for PSf, where it has been homogeneously dispersed without agglomeration and proven to contribute to the enhancement of antifouling activity [39]. Another mostly studied component as a filler material of the mixed matrix membrane, is GO. Graphene oxide is a two-dimensional (2D) material and hydrophilicity is its intrinsic property. Several studies reported successful dispersion of GO in a polymer solution and an improvement of antifouling activity and water flux [40,41].

Although the mixed matrix PSf membranes are extensively being studied for antifouling activity, they are limited to ultrafiltration membranes. There is a strong need to make PSf mixed matrix NF membranes. The most common process of NF membrane fabrication is interfacial polymerization (IP). In this process, the microporous polymer support membrane is first fabricated using the phase inversion process followed by the polyamide thin film created by a chemical reaction between the liquid–liquid interface [42]. However, this process has several limitations such as requiring multi-step work, being a time-consuming process, the requirement of a specific solvent, and a limited choice of polymer [43]. Due to the limited polymer choice for the IP method, the NF membrane cannot be prepared from PSf by this method. On the other hand, electrospinning is a versatile technique for the fabrication of nanofibrous porous membranes from a wide range of polymer. Currently, the electrospinning technique is being used to make a microfiltration/ultrafiltration membrane. There is a significant scope to study the electrospinning method to fabricate the NF membrane by optimizing the process parameters.

In this study, the removal of As, such as arsenite–As(III) and arsenate–As(V), was examined using a pressure-driven membrane process. The NF membrane was fabricated via the electrospinning of PSf with two different oxide materials, such as ZnO and GO. The resulting mixed matrix nanofibrous membranes were considered strengthened by GO and ZnO to avoid swelling or degradation during the operation with an antifouling tendency. The combination of the negative surface charge and small pore size should ensure a high removal of As(III) and As(V) by rejection in the water.

## 2. Materials and Methods

### 2.1. Materials

All chemicals used in the experiments were of reagent grade. Polysulfone (number average molecular weight, Mn ~22,000), N,N-dimethylacetamide (DMAC), acetone, zinc oxide (ZnO) nanoparticles, sodium (meta) arsenite (90%), and sodium arsenate dibasic heptahydrate (Na_2_HAsO_4_ 7H_2_O) were purchased from Sigma-Aldrich (Sydney, Australia). Graphene oxide water dispersion (0.4 wt%) was purchased from Graphenea (Cambridge, MA, USA). Deionized (DI) water was used for sample preparation and pure water flux measurements.

### 2.2. Membrane Preparation

The electrospinning solutions were prepared by dissolving 20 wt% PSf in a mixed solvent of DMAC and acetone in a 9:1 ratio at 40 °C. When GO and ZnO were used as filler materials, at first, their suspensions in the mixed solvent of DMAC and acetone were prepared, and then PSf was added into each solution, as mentioned in the process earlier. The weight% of the filler particles (GO and ZnO) was chosen from previous studies [39,41].

**Figure 1:**
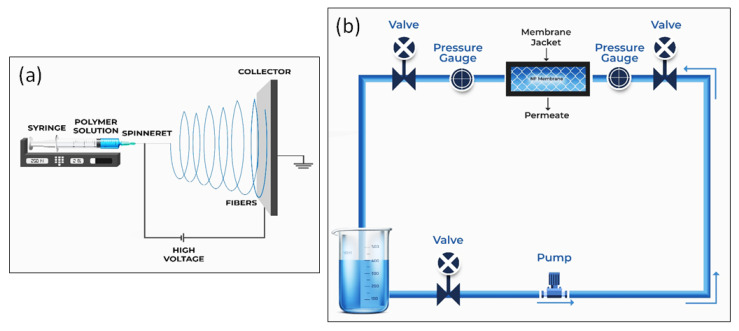
Schematic diagram of (**a**) electrospinning and (**b**) nanofiltration process [15].

The electrospinning was carried out inside a glove box. The setup is shown in Figure 1a. The electrospinning solution was loaded into a glass syringe (FORTUNA^®^ Optima) with interchangeable components from Sigma-Aldrich (Sydney, Australia), fixed with a size 18 SS dispensing metal needle (inner diameter of 0.84 mm). The needle was connected to a high-voltage supply, which could generate DC voltages up to 30 kV. The electrospun fibers were collected on a flat aluminum foil (collection screen) connected to the ground under the syringe. The flow rate of the solution was controlled by a syringe pump. The details of the electrospinning parameters used are provided in Table 1.

### 2.3. Characterization of Membranes

The conductivity of electrospinning solutions at room temperature was measured using the S220 SevenCompact benchtop pH/conductivity meter (Mettler Toledo, Melbourne, Australia).

The rheological properties of electrospinning solutions at 25 °C was analyzed using the HR-2 Discovery hybrid rheometer (TA Instruments, New Castle, DE, USA). The measurement was performed using a 60 mm cone (2.023°-cone angle) and plate geometry at a constant gap of 52 μm.

The surface morphology of the electrospun membranes was observed using the Nova NanoSEM 200 scanning electron microscope (FEI, Hillsboro, OR, USA). For sample preparation, membranes were cut into small pieces and coated (3 nm thickness) with Pt (to increase the conductivity before SEM imaging) using the Module Sputter Coater (SPI Supplies, West Chester, PA, USA). The fiber diameter distribution was estimated from these SEM images using ImageJ software, where at least 100 fibers were measured for each membrane.

The hierarchical structure of electrospun membranes was studied using the Quokka small-angle neutron scattering (SANS) (ANSTO, Sydney, Australia) [44] and Kookaburra ultra-small-angle neutron scattering (USANS) (ANSTO, Sydney, Australia) [45] instrument at ANSTO. The membranes were loaded into custom-built sample holders (20 mm diameter for SANS and 40 mm diameter for USANS) with a 1 mm path length filled with air as a background medium. The SANS data were collected in the scattering vector (q) range of 0.0007–0.1 Å^−1^, with source aperture to sample aperture distances of 2, 12, and 20 m, and a neutron wavelength (λ) of 5.0 Å^−1^ and 8.1 Å^−1^ (for lens optics), respectively. The USANS data were collected in the q range of 0.00004–0.001 Å^−1^, with λ of 4.74 Å^−1^. All the obtained SANS and USANS data were processed (data reduction, desmearing, and background subtraction) and combined as shown in our previous publications [46,47]. SasView (https://www.sasview.org/; accessed on 6 March 2020) analysis software with shape-independent model function fits was used to estimate the structural parameters of the samples.

The functional groups of electrospun membranes were measured using Spectrum One Fourier transform infrared spectrometer (FTIR) (Perkin Elmer, Boston, MA, USA) equipped with the attenuated total reflectance (ATR) accessory. The spectrum was collected in the transmission mode in the wavenumber range of 400–4000 cm^−1^ and with a resolution of 4 cm^−1^.

The phase structure of electrospun membranes was characterized using the D8 ADVANCE X-ray diffraction (XRD) (Bruker, Billerica, MA, USA) instrument. Data were collected in the 2θ range of 6° to 90° with a step size of 0.02 and counting time of 1 s.

The surface chemistry of electrospun membranes was analyzed using the K-Alpha X-ray photoelectron spectrometer (XPS) (Thermo Scientific, Waltham, MA, USA) with a monochromatic aluminum Kα X-ray source. The high-resolution XPS scans were conducted according to the peak being examined with a pass energy of 136.6 eV and a step size of 1.0 eV.

The specific surface area, average pore diameter, and pore area of electrospun membranes were measured with N_2_ as the adsorbate using the TriStar II 3020 surface area and porosity analyzer (Micromeritics, Norcross, GA, USA). Prior to analysis, the membranes were cut into very small pieces and degassed in a vacuum at 150 °C for 12 h by using a VacPrep™ 061 degasser (Micromeritics, Norcross, GA, USA). 

The static water contact angle of the electrospun membranes was measured (to determine the hydrophilicity) using the OCA20 contact angle goniometer (Particle and Surface Science, Filderstadt, Germany). The water droplet was placed on the membrane and the data recorded at 30 s. 

The zeta potential of the electrospun membrane was measured by the streaming potential method using the SurPASS electro kinetic analyzer (Anton Paar, Graz, Austria). For this purpose, each membrane was cut into two pieces of 20 mm × 10 mm size for the right and left sides of the adjustable gap cell, and zeta potentials were measured at different pH. In this method, 1.0 mM KCl solution was used as an electrolyte and 0.1 M HCl was used to change the pH of the electrolyte solution from pH 10 to 2 with the step-size of 1. All experiments were performed using triplicate samples and the values are reported as average with standard deviation.

### 2.4. Permeation Tests and Arsenic Rejection Experiments

The water permeability of electrospun membranes was determined by running deionized water and arsenite and arsenate solutions (prepared using respective salts at 40 ppb, as this is common in natural sources, e.g., ground water and surface water [5]) through the membranes (at 5 bar pressure) for 1 h using a crossflow filtration unit (Figure 1b). A membrane area of 0.0075 m^2^ was used for the tests conducted. The flow rate obtained was used in the Equation (1) to calculate the permeability of the membrane [48].

(1) $$L_{p}=\frac{Q}{A\Delta P}$$

where Q is the volume flow rate (L/h), A is effective membrane area (m^2^), ΔP is change in pressure (bar), and L_p_ is water permeability (L/(m^2^ h bar)).

Similarly, the rejection of arsenite and arsenate in water was evaluated at 5 bar pressure. The permeate was collected every 15 min for arsenate analysis, and finally reported as average. Arsenic concentrations were measured using Agilent Inductively Coupled Plasma Mass Spectrometer (ICP-MS). The rejection % of arsenite and arsenate was calculated using Equation (2) [49].

(2) $$\%R=(1-\frac{C_{p}}{C_{f}})\;\times \;100$$

where R is the rejection of arsenate (%) and C_p_ and C_f_ are the concentrations (ppb) of arsenate in the permeation and feed solutions, respectively. All water permeability and rejection experiments were performed in triplicate.

## 3. Results and Discussions

### 3.1. Characterization of the Electrospun NF Membranes

#### 3.1.1. Morphology Study

**Figure 2:**
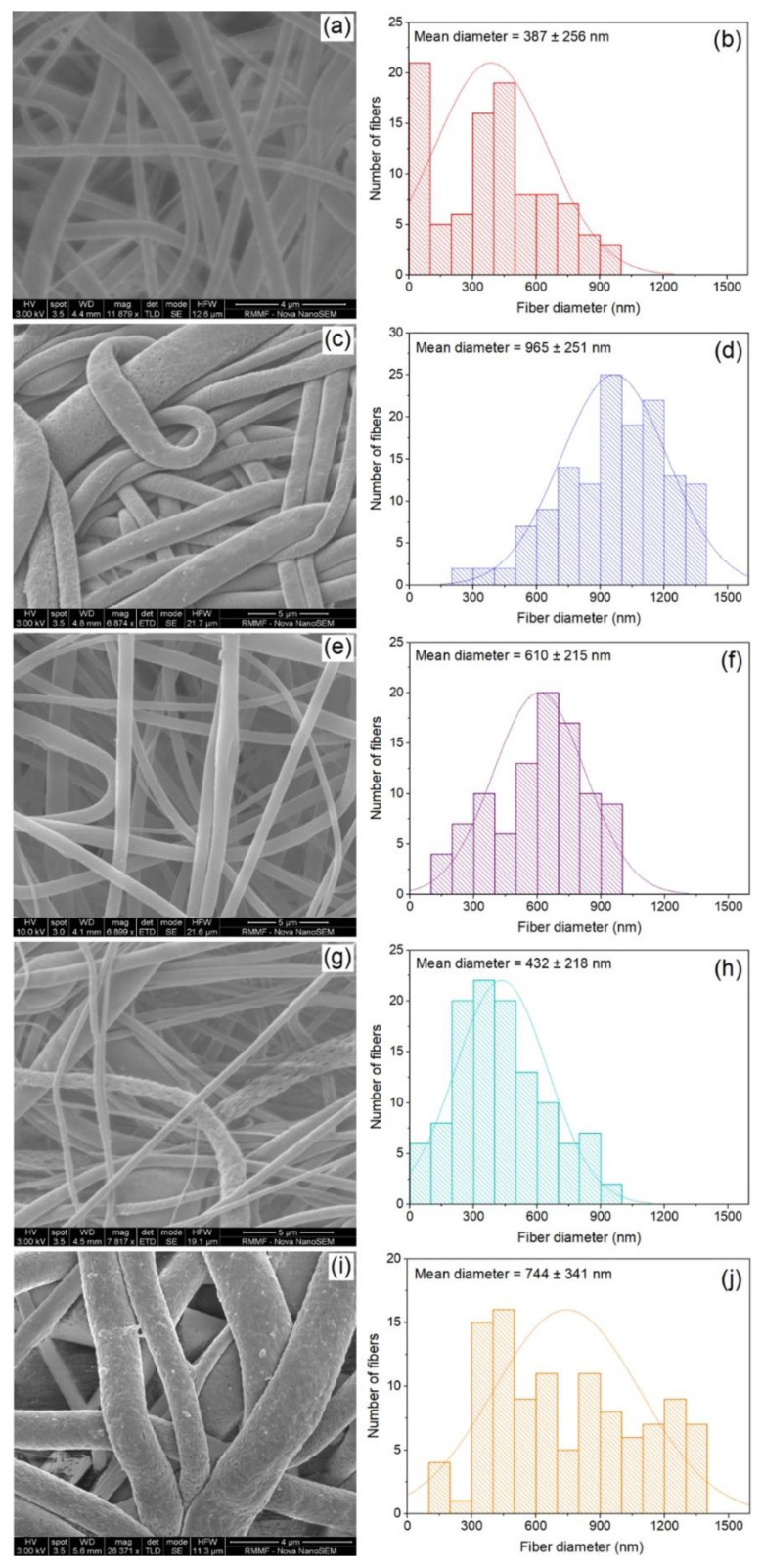
SEM images and fiber diameter distribution of fabricated membranes: (**a**,**b**) P, (**c**,**d**) GP-1, (**e**,**f**) GP-2, (**g**,**h**) ZP-1, and (**i**,**j**) ZP-2.

Figure 2 shows the surface morphology and fiber diameter distribution of electrospun P, GP-1, GP-2, ZP-1, and ZP-2 nanofibrous membranes from SEM. The analysis demonstrated that the addition of filler material has a profound impact on the surface and diameter of the fiber. The PSf fiber (thereafter simply mentioned as sample P) surface was smooth and randomly aligned with no beads. No beads were observed even after GO addition, which indicates stable fiber formation [50]. The fiber surface of GP-1 exhibits a nano-porous structure. Like the GP-1 membrane, ZnO incorporation in the membrane resulted in the formation of a nano-porous fiber surface. The magnified image exhibited that ZP-2 had a more porous structure. In the mixed matrix membrane, the nanogap in the polymer matrix, created by the addition of the filler particle, defines the pore size of the membrane. The membrane with more ZnO is more porous, presumably because of the nanogap area formed due to the well dispersion of ZnO [39,51]. The weak interaction between the ZnO and polymer matrix may also contribute to making porosity around the filler material [52]. On the other hand, with increasing loading of GO in GP-2, the porosity was not observed on the fiber surface. This could be due to the segregation of filler particles from the polymer matrix because of higher loading [52].

For P, a stable jet was obtained at 15 kV and the resulting average fiber diameter is 387 ± 265 nm, which is in line with results reported earlier [53,54]. Loading of GO in PSf matrix increased the fiber diameter for GP-1 and GP-2 than that of P. Although the operation voltage of GP-1 is lower than GP-2, the average fiber diameter of GP-1 is higher [55]. With the increasing graphene loading in GP-2, the solution viscosity and conductivity was increased. Although the required voltage to obtain the stable jet was higher because of increasing viscosity, the increased conductivity of the solution is critical to obtain a lower diameter of the fiber.

For ZP-1, a significant change in fiber diameter was not observed after the ZnO incorporation, as the stable jet was obtained at a lower voltage of 13.5 kV because of the higher conductivity of the ZP-1 dope solution (241 mS/cm) than pristine polymer solution (177 mS/cm), which decreases the minimum required applied voltage [56,57]. On the other hand, increasing the ZnO loading in ZP-2 resulted in increasing the mean diameter of fiber by almost double the pure PSf electrospun nanofiber, as a higher required voltage of 17.5 kV was dominating to define the property of the fiber [58,59].

#### 3.1.2. SANS and USANS Study

**Figure 3:**
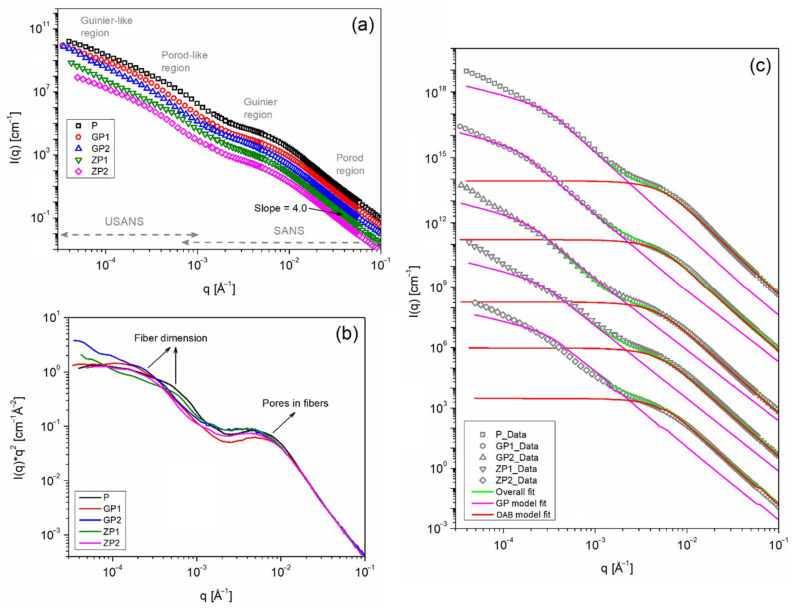
(**a**) Combined SANS and USANS intensity profile and (**b**) Kratky plot of the five membranes. (**c**) Model function fits. The intensity in graph (**a**,**c**) are offset for clarity.

Figure 3a shows the measured SANS and USANS intensity profiles of the fabricated membranes. The measured SANS and USANS data provide hierarchical structural information of membranes in the size range of 1 nm to 20 µm. The neutron scattering length density (SLD) of polysulfone (1.87 × 10^−6^/Å^2^), GO (1.87 × 10^−6^/Å^2^), and ZnO (4.77 × 10^−6^/Å^2^), calculated using the NIST online calculator (https://www.ncnr.nist.gov/resources/activation/; accessed on 6 March 2020), provides good contrast (i.e., scattering intensity) against the background. The scattering profile exhibited a Porod region at high q (0.02 < q < 0.1 Å^−1^), a Guinier region at mid-q (0.002 < q < 0.02 Å^−1^), and a further Porod-like (0.0002 < q < 0.002 Å^−1^) and a Guinier-like (0.00004 < q < 0.0002 Å^−1^) regions at low-q. A slope value of 4.0 obtained from the power-law fit in the high-q region indicates the membranes exhibit a sharp or smooth interface with the surrounding medium, which can be attributed to the hydrophobic nature of polysulfone [60]. A strong upturn with no clear Guinier region was observed at the very low-q region, which describes the structure of the membrane to extend beyond the USANS length scale. The broad feature observed at low-q and mid-q regions can be attributed to the electrospun fiber dimension and nanopores in the fibers, as observed in SEM. This is more evident from the Kratky plot (Figure 3b), which shows the features very clearly by dividing out the decay of the scattering [60].

A combined form factor model function, i.e., shape-independent Debye-Anderson-Brumberger model (high-q to mid-q) + Guinier-Porod model (mid-q to low-q) was used to fit the scattering data and estimate the structural parameters of the membranes. Figure 3c shows the model fit results. The Debye-Anderson-Brumberger (DAB) model describes scattering from the randomly distributed two-phase system with smooth interfaces, and provides information on correlation length (i.e., measurement of the average spacing between regions) [61]. On the other hand, the Guinier–Porod (GP) model estimates the dimensionality and size of the scattering objects, such as spheres, rods, platelets, and intermediate structures [62]. The DAB model fit returned a nanopore value of around 35 nm for the polysulfone membrane, which increased to around 39 nm and 43 nm for GP1 and GP1 membranes, respectively, which indicate an increase in porosity with increase in GO. ZP1 and ZP2 membranes exhibited an increased pore value around 41 nm and 43 nm, respectively. The GP model was fit with a dimensional variable (S) value of 1 (corresponding to rods), and the Porod slope and radius-of-gyration (Rg) was estimated. The fits returned a Porod slope value of 3.8 for the polysulfone membrane, which indicates the electrospun membrane surface to have a surface fractal nature [60]. The values are marginally reduced to 3.5 and 3.6, respectively for GP and ZP membranes, which indicates an increase in porosity of the polysulfone membrane with an addition of GO and ZnO. The GP fit also returned an Rg value of ~380, ~760, ~610, ~430, and ~640 for P, GP1, GP2, ZP1, and ZP2 membranes, which are in general agreement with fiber diameters estimated from the SEM. The observed differences could influence the surface properties of fabricated membranes.

#### 3.1.3. FTIR Analysis

**Figure 4:**
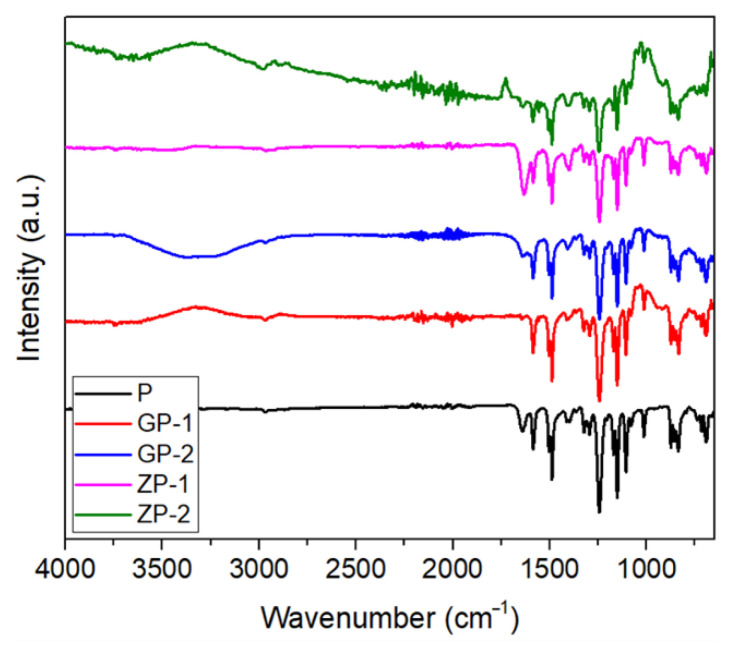
FTIR spectrum of fabricated membranes.

FTIR spectra were scanned for the five synthesized membranes of different compositions (Figure 4) and major peaks were listed in Table 2 for easy understanding of the peak shift. The absorption peak at 1149 cm^−1^ represents the O=S=O stretching vibration of pure PSf [63]. The asymmetric stretching of the C–O–C group is denoted by the absorption band at 1241 cm^−1^ [64]. On the other hand, the 1584 cm^−1^ peak corresponds to the C=C bond of the benzene ring. The CH_3_ stretching vibration is denoted by the peak, at 2972 cm^−1^ [65]. 

In ZP-1 and ZP-2, the characteristic peak of PSf remains, although little changes in the peak intensity were observed with a slight peak shift of C–O–C in ZP-2 and C–H in both ZP-1 and 2; this could be due to the interfacial bonding between PSf and ZnO. The characteristic peak of ZnO is observed at 588 and 1118 cm^−1^ ZP-1 and ZP-2 after close observation. As most of the characteristic peaks of the pristine PSf-like benzene ring, O–S–O etc were not shifted in ZP-1 and ZP-2 after the addition of ZnO, and the hydrogen bond formation cannot be confirmed from the FTIR results, which is aligned with other reports on the ZnO/PSf membrane [39]. It also can be concluded that there was no covalent bond formation as the new peak was not observed.

Like ZP-1 and ZP-2, after GO addition to the PSf matrix, the characteristic peaks of pristine PSf are observed with a slight peak shifting of O–S–O in GP-2 and C-H in GP-1,2 due to the interfacial bonding between the PSf fiber matrix and GO particles. However, a broadband is observed between 3000 to 3500 cm^−1^ in the FTIR spectrum of GP-1 and GP-2, which is attributed to the stretching mode of the –OH functional group of GO. The peak observed at 1640 cm^−1^ corresponds to the C=O stretching mode of the carboxylic acid functional group. Peak shifting of the –OH functional group was also observed with two different loading amounts of GO as a more interfacial bond formed. Two other peaks of GO were observed at 1403 and 1104 cm^−1^, respectively, which attributed to C–OH and C–O bands, respectively.

#### 3.1.4. XRD Analysis

**Figure 5:**
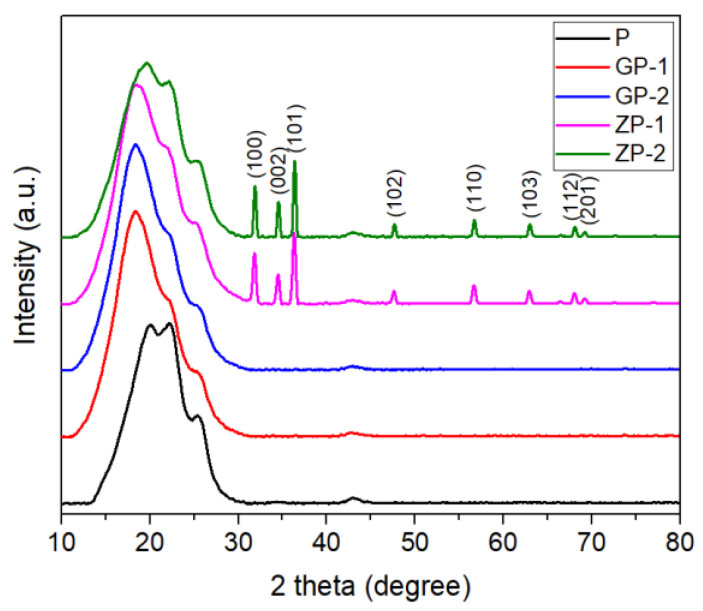
XRD graphs of fabricated membranes.

The XRD analysis of GP-1 and GP-2 has been shown in Figure 5. The phenyl groups of the PSf molecular structure limit the ordered stacking of its chains and result in making an amorphous structure that is indicated by the characteristic peaks of 7.5° and 20° [66,67]. The characteristic peak of GO is observed at 10°, which indicated the ordered structure of GO [67]. Although the diffraction pattern of GP-1 and GP-2 is very similar to that of PSf, the 7.5° and 20° peaks were shifted to smaller angles because of the GO content. This observation indicates that the GO incorporation causes tailoring of polymer entanglement to slightly order the structure. The effect of GO on the polymer structure in the mixed matrix system is also reported by several studies [68]. There was no specific peak observed in GP-1 or GP-2 for GO, which is ascribed to the low GO content relative to the polymer. A similar result was reported by other studies [67,69].

The XRD analysis of ZP-1 and ZP-2 has been presented in Figure 5. The peaks at 31.9°, 34.54°, 36.32°, 47.6°, 56.65°, 62.93°, 66.47°, 68.06°, and 69.1° were obvious in the XRD spectra of ZP-1 and ZP-2, as these are the characteristic peaks of ZnO. The 7.5° peak intensity of PSf decreased after ZnO incorporation due to the interfacial bonding between the PSf fiber matrix and ZnO filler materials. The 20° peak was shifted to a lower value. The peak shifting of PSf could indicate that the structural order of PSf was increased in the presence of ZnO in the polymer matrix [67].

#### 3.1.5. XPS Analysis

**Figure 6:**
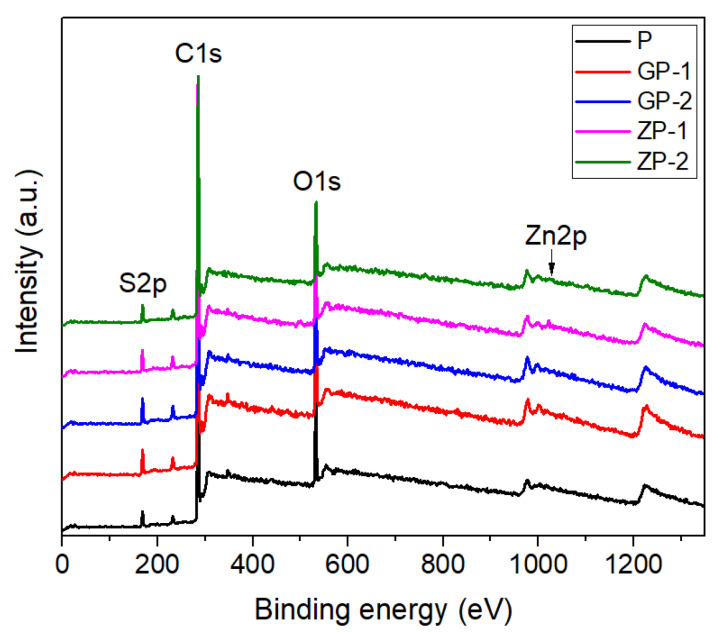
XPS spectra of fabricated membranes.

Multiplexed spectra were obtained from XPS for carbon, oxygen, zinc, and sulfur. In the survey scan, the membranes (Figure 6) show photoelectron lines at a binding energy of about 284.4, 531.4, and 167.5 eV attributed to C1s, O1s, and S2p, respectively. All these peaks have been observed in all five membranes due to the matrix in PSf containing C, O, and S elements [26]. In the case of ZP-1 and 2, the additional peak for Zn2p is observed at 1022 eV, which is attributed to oxide bonds.

From the atomic weight percentage of each element and compositions listed in Table 3, which is calculated using Casa XPS from Figure 6, the presence of the filler material is confirmed. With the addition of GO and ZnO, the oxygen percentages in the membranes have increased. In the case of GP 1 and 2, there are no additional elements from P as GO contains C and O only, which are already present in polysulfone polymer. The other two membranes ZP 1 and 2 have Zn as the additional element, which confirmed the presence of ZnO in the PSf matrix. In ZP-1 and 2, %C decreases with the increase in ZnO, but %O increases as expected.

**Figure 7:**
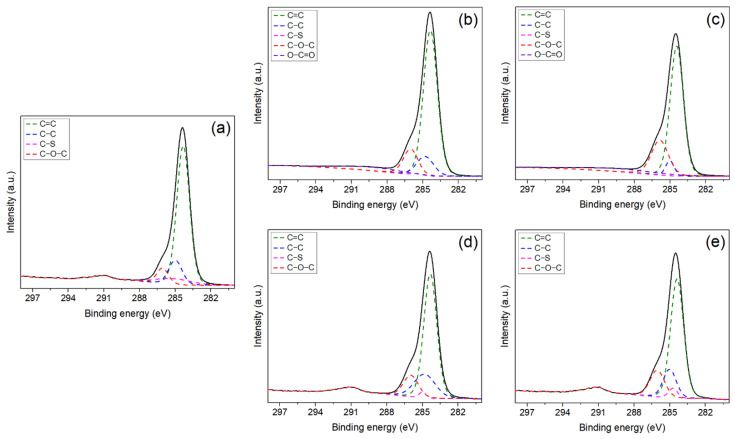
High-resolution C1s XPS spectra of fabricated membranes: (**a**) P, (**b**) GP-1, (**c**) GP-2, (**d**) ZP-1, and (**e**) ZP-2.

High-resolution C1s XPS spectra (Figure 7) were acquired for all the experimental membranes and deconvoluted into sub-peaks representing various carbon functionalities, i.e., C=C, C–C, C–S and C–O–C at 284.4, 284.8, 285.5, 286, and 289 eV, respectively, to calculate the distribution of carbon functionalities presented in Table 4. The binding energies of C 1s, O 1s, Zn 2p3, and S 2p remained unaltered, even though the relative contribution of different oxidation states of each element changes. There was no noticeable change in the peak position of all four carbon functionalities in the synthesized five membranes, which confirms no new chemical bond formation or reactions happened during the addition of the filler particles (GO and ZnO).

In GP-1 and GP-2, the C–S ratio decreased to a very low level (<1%) with the addition of GO compared to P (8.1%). The characteristic peak of O–C=O is present in GP-1 (7.5%) and GP-2 (9.3%), as they contain GO at 289 eV [70,71], which is higher in GP2, confirming a higher amount of GO content. The ratio of C–O–C increased in GP-1 and 2, as GO has this functionality as well [70,71]. The increased ratio of C-O-C is also observed in GP-2 (22%) versus GP-1 (12.6%), which confirms a higher % of GO in GP-2.

#### 3.1.6. Brunauer–Emmett–Teller (BET) Surface Area Analysis

**Figure 8:**
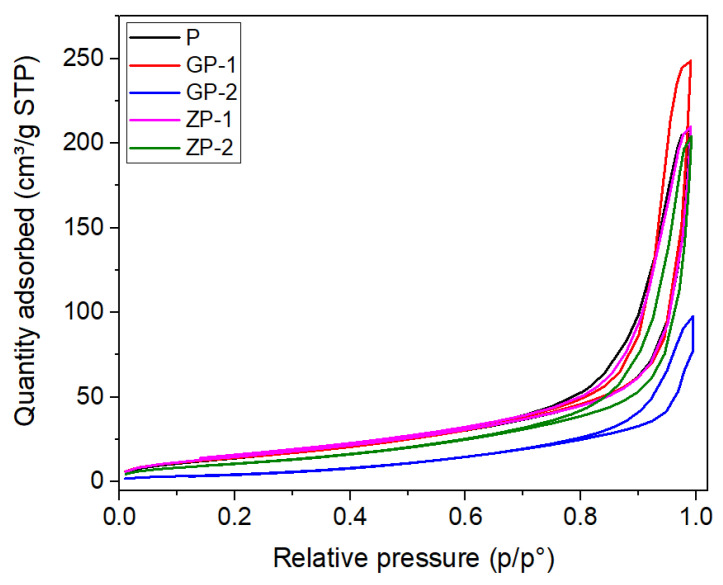
Nitrogen adsorption–desorption isotherms of fabricated membranes.

A N_2_ adsorption–desorption isotherm has been constructed from the Brunauer–Emmett–Teller (BET) surface area measurement and is depicted in Figure 8 for P, GP-1, GP-2, ZP-1, and ZP-2, respectively. Specific materials have specific isotherm qualities and modes of gas adsorption and desorption. The isotherms presented can be categorized as type IV due to the significant hysteresis loop, which means the membranes are mesoporous. The observed BET surface area (Table 5) of P is 55.1 m^2^/g, which is higher than 0.051 m^2^/g that was reported previously for 15 wt% PSf in 1-methyl-2-pyrrolidone (NMP) solvent [72].

The BET surface area (Table 5) of P (55.1 m^2^/g) decreases significantly to 20.87 m^2^/g in the case of GP-1 and 6.39 m^2^/g for GP-2. The addition of GO in the PSf matrix leads to a decreased surface area, which is more with a greater amount of GO loading. In the case of ZP-1, the BET surface area is almost the same as P, but when the ZnO loading increased to 1%, which is double of 0.5%, the BET surface area is decreased remarkably to 16.82 m^2^/g. The pore surface area of the membranes is higher than BET surface area for all the five membranes, as these are the nanofibrous membranes consisting of nanofibers of 200 to 400 nm diameter. 

The membranes have 57.09, 77.76, 86, 52.98, and 78.92% porosity in P, GP-1, GP-2, ZP-1, and ZP-2, respectively, with a pore size of 0.65, 1.83, 1.75, 0.66, and 1.71 nm. The porosity % increases with the addition of filler materials and the increase in applied voltage [73,74,75]. The same pore size could be due to similar fiber diameter and porosity % developed during electrospinning conditions. The pore size below 2 nm has confirmed the synthesis of NF membranes, which tend to have a higher removal rate of arsenite.

#### 3.1.7. Contact Angle and Surface Charge Analysis

**Figure 9:**
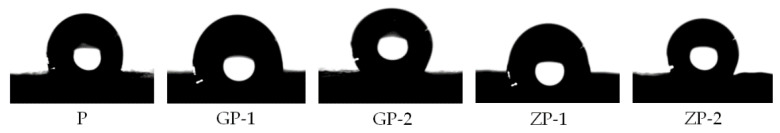
Water contact angle images of fabricated membranes.

Membrane surface hydrophilicity is an important factor in determining the membrane flux and membrane performance [76]. Higher membrane surface hydrophilicity means higher permeation flux and higher anti-fouling performance [49]. The hydrophilicity of the membranes is measured by the water contact angle and listed in Table 5. Polysulfone membranes are hydrophobic with a water contact angle of 125°, which stays the same and is following the same trend as reported previously by Arribas et al. [77], who reported the water contact angle in the range of 116 to 129°. Compared to the pure PSf membrane with a 0.01% GO-loaded PSf membrane, the water contact angle decreases from 125 to 100° and decreases further to 97° with the increase in GO loading to 0.1% (Figure 9). This is due to the increase in the hydrophilic groups on the membrane surface, which result in a decreased surface tension with the water [78]. Thus, it can be summarized that the hydrophilicity of electrospun PSf membranes could be increased by adding GO to the fiber matrix, which can address the hydrophobic nature of the PSf membranes [79]. With the addition of 0.5% ZnO, the contact angle of the electrospun PSf membrane also slightly decreases by 1° to 124°, but with a further decrease of 5° with the increase in the loaded ZnO in PSf to 1% (Figure 9). Therefore, the addition of ZnO in the PSf nanofiber matrix also increases the hydrophilicity, which is required for water filtration [73,74], as ZnO increases the surface energy, resulting in an easier spread of water on the membrane surface [75].

**Figure 10:**
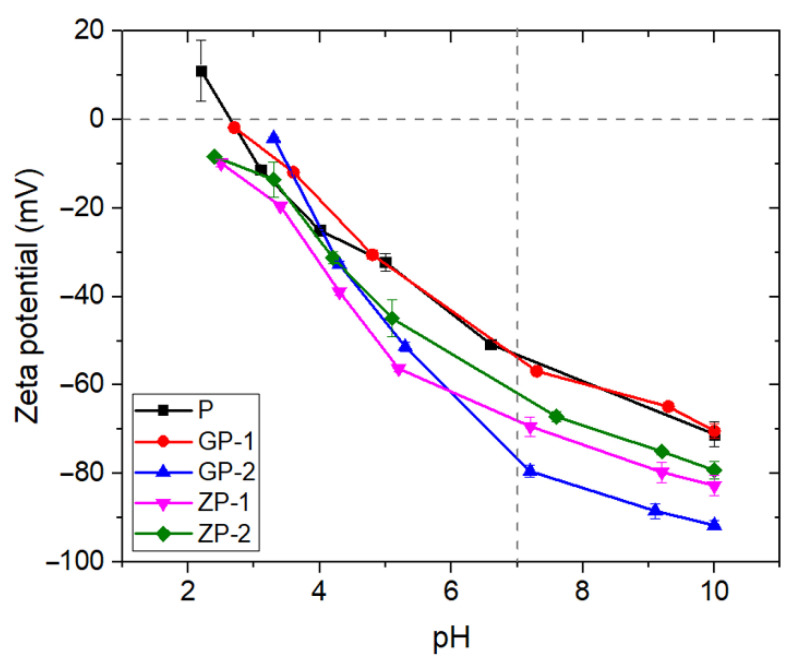
The surface zeta potential of fabricated membranes.

The change in the membrane surface chemistry and the fouling tendency after the addition of filler materials can be understood by studying the zeta potential of the membrane. The streaming potential was measured by using the electro-kinetic analyzer to determine the surface zeta potential of the membranes in this study. Isoelectric point (the pH value where no charge is present at the surface) was used to define the changes in the membrane surface charge due to the GO and ZnO addition to the fiber matrix. The surface zeta potential of the synthesized membranes as a function of pH value is shown in Figure 10; the values support the previously reported results [80]. The polymer electrospun membrane P, where no filler materials were added, has an isoelectric point of 2.6. The membrane is positively charged at low pH (below ~2.6) and negatively charged at high pH as well as neutral. Specific ionic adsorption is the only process possible for surface charge formation of the pristine P membrane, as PSF has no dissociable functional groups [81]. All the other membranes did not demonstrate any positive zeta potential values within the measured pH range. The addition of GO and ZnO in the PSf fiber matrix results in a negative shift in the surface zeta potential curve and decreases the membrane surface isoelectric point as well due to the presence of additional ions such as the carboxyl functional group in GO in the case of GP-1 and 2, whereas it is the Zn^2+^ ion in the case of ZP-1 and 2. The isoelectric point is decreased to 2.5 for the loading of 0.01% of GO, when it shifts to almost the same for 0.1% of GO. Thus, not a major change with the change of GO loading. However, ZP-1 and ZP-2 membranes were entirely negatively charged within the studied pH range, with no isoelectric point that could be evaluated within the experimental pH range. It indicates a highly negatively charged membrane surface desired for As(V) removal by the Donnan Exclusion mechanism.

### 3.2. Evaluation of Synthetic NF Membranes on the Rejection of Arsenic

#### 3.2.1. Water Permeability of Membrane

The water flux was measured in filtration experiments for both water and arsenic-contaminated water (Table 6). The water permeability is lower in the case of the arsenic-contaminated water than pure water for all the membranes for the presence of arsenic. Notably, in case of GP-1 and ZP-2, the water permeability is same for pure water and As(III)-contaminated water. Except for the ZP-1 membrane, all the membranes have lower water permeability for As(V)-contaminated water compared to As(III)-contaminated water.

The water permeability is highest for P among these five membranes. With the loading of filler materials, the water permeability decreases significantly, as shown in Table 6. It is ascribed to the change of the membrane and its surface properties, such as hydrophilicity, membrane surface charge, pore size, % porosity etc., because of the addition of GO and ZnO.

Among GP-1 and 2, GP-1 has a slightly higher permeability for As-contaminated water due to the larger pore size, whereas GP-2 has a higher permeability for pure water for the higher porosity %, as they have a similar hydrophilic nature. In the case of ZP-1 and 2, ZP-2 has higher water permeability, as ZP-2 has nearly 26% more porosity than ZP-1. Higher permeability is favorable for a higher arsenic rejection rate [82].

#### 3.2.2. Rejection of As(III) and As(V)

Following the same trend as water permeability, the arsenite rejection for P and ZP-2 is highest at 27% (Table 6), which has a lower pore size and higher % of porosity on the membrane surface favoring the arsenite removal. The addition of ZnO results in decreasing the arsenite removal to 8% initially and again increasing to 27% with the addition of 1% ZnO, as the porosity % increased with the increase of ZnO %, and the pore size becomes bigger but remains within 2 nm. From Table 6, it is clearly shown that the arsenite removal % decreases with the addition of GO and lowers with the higher loading amount of GO, as it is reported 27, 24, and 19% for P, GP-1, and GP-2, respectively. The reason for the mentioned outcome is the increment of pore size and porosity % of the PSf membrane, which are the key factors for arsenite removal, as it follows the steric exclusion mechanism [15]. There is no report yet on the arsenite removal by electrospun membranes, but recently, a 99% arsenite removal rate has been achieved by the polyamide intercalated nanofiltration membrane [15,83]. In this case, pore size and porosity % play the main roles, as the pH of arsenite solution was between 6.4 to 7.4 and arsenite stays in water as uncharged H_3_AsO_3_ in this pH range [84].

The dominant factor for arsenate rejection is the membrane surface charge, as it follows the Donnan exclusion mechanism [15,85]; the arsenate solution pH was kept in between 5.3 and 6.9, and in this pH range the arsenate behaves as negatively charged H_2_AsO_4_^−^ (pH 2–6) and HAsO_4_^2−^ (pH 6–11.5) [84], but the steric effect also plays a role [86]. The arsenate rejection of P, GP-1, GP-2, ZP-1, and ZP-2 are shown in Table 6 as 43, 13, 60, 17, and 71%, respectively. It is clearly shown that with the addition of filler materials such as GO and ZnO, they initially cause a decrease in arsenate rejection but increase later with the addition of a higher amount following the trend of previous reported results by Rezaee et al. [78]. The negative surface charge increased with the addition of GO and ZnO, which results in a high arsenate rejection rate and the membranes have a negative surface charge within the studied pH range, which is required for arsenate rejection. GP-2 and ZP-2 rejected 60 and 71% of arsenate, which is higher than the only reported result on electrospun membranes for arsenate removal by Bahmani et al. [82], where GP-1 and ZP-1 demonstrated only 13 and 17% of arsenate rejection. This could be because of the porosity % and hydrophobic nature.

A little trade-off effect between water flux and rejection rate of arsenic has been observed (Table 6). The reason behind this could be due to porosity and surface charge in the case of arsenic rejection, whereas porosity is the only driving factor for permeability.

## 4. Conclusions

In the present study, nanofiltration composite membranes of PSf with GO and ZnO were fabricated by an electrospinning process. The addition of oxide materials to PSf increased the fiber diameter and nanopore size in fibers, whereas they decreased the BET surface area and isoelectric point of the membranes. Moreover, both the oxide materials increased the surface negative charge of the fabricated membranes and enhanced their hydrophilicity. The water permeability of the five membrane systems for three different water systems is in the range of 0.1 to 15 L h^−1^ m^−2^ bar^−1^. The increased negative charge on the membrane results in a higher removal rate of arsenate for GP-2 (60%) and ZP-2 (71%) composite membranes compared to the pristine P (43%) membrane. However, with the addition of GO and ZnO in the PSf matrix, the arsenite removal rate was observed to decrease by one third to 8% from 27%, except with the same for ZP-2 as P. Therefore, electrospun mixed matrix membranes removed arsenate more effectively due to the Donnan exclusion effect, where the membranes exhibit negative charges (induced by GO and ZnO) and the arsenate stays in the form of negatively charged ions in the pH range of 2–11.5. The developed mixed matrix composite membranes could potentially be applied for the effective removal of arsenate from water.

## Figures and Tables

**Table 1:** Electrospinning parameter and solution properties used for membrane fabrication.

Membranes	Polymer	Filler	Applied Voltage (kV)	Flow Rate (mL/h)	Distance between Collector and Needle Tip (cm)	Conductivity at 25 °C (mS/cm)	Viscosity at 25 °C (Pa.s)
P	20% PSf	---	15	1.5	15	177	1.608
GP1		0.01% GO	15	1.5	15	111	1.48
GP2		0.1% GO	17.25	1.5	15	186	1.887
ZP1		0.5% ZnO	13.5	1.5	15	241	1.137
ZP2		1% ZnO	17.25	1.5	15	230	1.316

**Table 2:** Major FTIR peaks and corresponding functional groups of fabricated membranes.

Functional Group	Peak Position in Terms of Wavelength (cm^−1^)				
	P	GP-1	GP-2	ZP-1	ZP-2
–OH	---	3000	3310	---	---
C=O	---	1643	1637	---	---
Benzene ring	1584	1584	1584	1584	1584
C–OH	---	1403	1403	---	---
C–O–C	1241	1241	1241	1241	1244
O–S–O	1149	1149	1152	1149	1149
C–O	---	1104	1104	---	---
C–H	1013	1015	1015	1015	1012
C–H	834	834	834	834	834
Zn–O	---	---	---	588	591
Zn–O	---	---	---	1118	1118

**Table 3:** Element composition of fabricated membranes obtained from XPS data.

Elements (Atomic wt%)	P	GP-1	GP-2	ZP-1	ZP-2
C	84.9	84.8	84.5	83.5	83.2
O	12.4	12.2	11.9	12.8	13.0
S	2.7	3.1	3.6	3.4	3.6
Zn	N/A	N/A	N/A	0.3	0.2

**Table 4:** Quantification of deconvoluted XPS C1s spectra of fabricated membranes.

Deconvoluted Peaks (Atomic wt%)	P	GP-1	GP-2	ZP-1	ZP-2
C=C	71.7	68.3	63.6	65.2	64.7
C–C	13.1	11	4.8	20.3	15.5
C–S	8.1	0.6	0.4	1.6	3.7
C–O–C	7.1	12.6	22	12.9	16.1
O–C=O	---	7.5	9.3	---	---

**Table 5:** Surface properties of fabricated membranes.

Membrane	Average Fiber Diameter (nm)	Surface Area (m^2^/g)		Porosity (%)	Pore Size (nm)	Contact Angle (°)
		BET	Pore			
P	387	55.1	73.3	57.09	0.65	125 ± 2
GP-1	251	20.88	72.98	77.76	1.83	100 ± 3
GP-2	216	6.39	39.27	86	1.75	97 ± 2
ZP-1	218	57.65	64.96	52.98	0.66	124 ± 1
ZP-2	341	16.82	62.95	78.92	1.71	119 ± 6

**Table 6:** Filtration performance of fabricated membranes.

Membrane	Water Permeability (L hr^−1^ m^−2^ bar^−1^)			As(III) Removal (%)	As(V) Removal (%)
	Pure Water	As(III)- Contaminated Water	As(V)- Contaminated Water		
P	15	8	7	27	30
GP-1	1	1	0.8	24	13
GP-2	1.3	0.8	0.4	19	60
ZP-1	0.3	0.1	0.1	8	17
ZP-2	13	13	11	27	71

## Data Availability

The datasets generated for this study are available on request to the corresponding author.
